# 3D CT–Based quantification of airway structural changes during asthma exacerbations: a retrospective observational study

**DOI:** 10.1186/s12890-026-04464-z

**Published:** 2026-06-30

**Authors:** Ryosuke Ozoe, Kenji Mizumura, Yosuke Nemoto, Michio Kawamura, Shun Yokota, Naho Furusho, Yusuke Kurosawa, Shiho Yamada, Masayuki Nomoto, Yuko Iida, Go Naka, Yutaka Kozu, Yoshiko Nakagawa, Takashi Oki, Tetsuo Shimizu, Motoyasu Iikura, Shuichiro Maruoka, Yasuhiro Gon

**Affiliations:** Division of Respiratory Medicine, Department of Internal Medicine, Nihon University School of Medicine, 30-1 Oyaguchi Kami-cho, Itabashi-ku, Tokyo, 173-8610 Japan

**Keywords:** Semi-automated 3D-CT analysis, Asthma, Bronchial endpoint count, Bronchial volume, Clinical relevance, Exacerbations

## Abstract

**Background:**

Traditionally, mucus plugs have been evaluated using two-dimensional computed tomography (CT) images through expert visual assessment, a process that requires substantial experience and specialized knowledge of airway anatomy. In the present study, we propose a novel approach employing three-dimensional CT (3D-CT) with semi-automated quantification, which can be performed in routine clinical practice without requiring expert readers. We aimed to demonstrate its feasibility and clinical relevance for assessing mucus plug burden and exacerbation severity in asthma.

**Methods:**

This exploratory, single-center, retrospective study included 23 patients with asthma and a history of exacerbations. Using a clinically available 3D-CT application, we semi-automatically quantified bronchial endpoint count and bronchial volume in both stable and exacerbation phases, and calculated their percentage change. We further assessed correlations of these parameters with mucus plug scores and arterial blood gas measurements.

**Results:**

The bronchial endpoint count and bronchial volume were significantly lower during exacerbations than during the stable phase; both showed a significant negative correlation with mucus plug scores during exacerbations. The percentage change of bronchial endpoint count and bronchial volume from the stable to exacerbation phase also correlated with mucus plug scores. The bronchial endpoint count and bronchial volume at exacerbation-related admission did not correlate with the PaO_2_/FiO_2_ (P/F) ratio; however, their percentage change from the stable phase to exacerbation-related admission significantly correlated with the P/F ratio.

**Conclusions:**

Clinically available semi-automated 3D-CT analysis enables objective and reproducible quantification of bronchial endpoint count and bronchial volume during asthma exacerbations, eliminating the need for expert readers. Their percentage change from the stable to exacerbation phase not only reflect mucus plug burden but also correlate with oxygenation status. These imaging biomarkers enable objective and quantitative assessment of structural airway abnormalities, including airway narrowing, distal airway obstruction, and mucus impaction. They are not intended to replace conventional clinical or physiological assessment, but rather to provide complementary structural information regarding the underlying causes of physiological abnormalities, such as impaired oxygenation, when detected by physiological evaluation. Furthermore, such quantitative structural assessment may contribute to a better understanding of airway structural changes and airway dynamics during asthma exacerbations.

**Supplementary Information:**

The online version contains supplementary material available at https://doi.org/10.1186/s12890-026-04464-z.

## Background

Bronchial asthma, a chronic inflammatory airway disease, is predominantly driven by type 2 inflammation. It results in airway smooth muscle hypertrophy, luminal narrowing, and hypersecretion of mucus, collectively manifesting as clinical symptoms such as cough and dyspnea [1–6]. Asthma affects an estimated 262 million individuals worldwide, with both its prevalence and incidence showing a continuing upward trend [7]. Acute exacerbations of asthma can be life-threatening events that require timely and accurate clinical decision-making. In routine clinical practice, exacerbation severity is assessed using a comprehensive approach that includes symptom evaluation, physical examination, pulmonary function testing, and peripheral oxygen saturation measurement. Conversely, although imaging modalities such as chest computed tomography (CT) are routinely employed to differentiate comorbid conditions, including pneumonia, they are not conventionally used to directly assess asthma exacerbation severity [8, 9].

Recently, imaging analysis technologies have advanced remarkably, and there have been various attempts at applying these techniques in the context of asthma. In contrast, CT is routinely used for differential diagnosis and evaluation of airway remodeling in patients with asthma, and there is growing interest in utilizing imaging diagnostics as biomarkers [10]. As a complement to conventional blood, exhaled breath, and pulmonary function tests, the use of CT has also been suggested for evaluating airway structural changes [10].

However, these findings have been primarily described during the clinically stable phase, and the structural changes that occur during asthma exacerbations—and their clinical significance—remain incompletely understood.

Furthermore, constructing and analyzing three-dimensional images from CT scans (3D-CT) has become feasible in clinical practice. In the field of respiratory medicine, 3D-CT facilitates detailed visualization of the lung lobes, bronchial trees, and pulmonary vasculature and enables quantitative assessment of bronchial endpoint counts (defined as the number of visible distal airway branches), bronchial volumes (reflecting the luminal size of the bronchial tree), and airway wall thickness. This allows for a more comprehensive anatomical and functional analysis than when using conventional CT. Indeed, volumetric analysis using 3D-CT has demonstrated excellent accuracy in predicting postoperative pulmonary function, and in patients with chronic obstructive pulmonary disease, 3D-CT–based phenotypic classification has been correlated with clinical characteristics [11, 12]. In the context of severe asthma, 3D-CT analysis employing airway quantification software has also proven valuable in evaluating treatment responses to bronchial thermoplasty [13]. Collectively, these findings highlight the potential utility of 3D-CT in elucidating disease pathophysiology and informing therapeutic decision-making in various respiratory disorders. However, to the best of our knowledge, in no studies have airway structural alterations during asthma exacerbations been quantitatively assessed using 3D-CT, nor have their associations with clinical parameters been explored.

Recently, the significant role of mucus plugs in asthma exacerbations and disease severity has been increasingly recognized [14–17]. Mucus plugs are primarily composed of mucins, water, and inflammatory cells, and their formation is believed to result from excessive mucus production and impaired mucociliary clearance [18, 19]. In asthma, the overexpression of MUC5AC, a major gel-forming mucin, leads to the formation of highly viscous and cohesive mucus plugs as mucin glycoproteins aggregate through disulfide bonding [20]. Moreover, the involvement of inflammatory cells, especially eosinophils, enhances the viscosity and gel-like consistency of mucus, thereby impairing its clearance [21–23]. In fact, mucus plugs reportedly persist in the airways of patients with asthma for extended periods and contribute to sustained airflow obstruction [14]. In addition, during exacerbations, marked narrowing of the airway lumen in the fourth- and fifth-generation bronchi has been observed, accompanied by an increased prevalence of mucus plugs [15, 24]. Despite these associations, the current standard method for mucus plug evaluation—the mucus plug score—relies on manual visual assessment of CT images by experienced radiologists or pulmonologists. This approach demands specialized expertise and is time-intensive, which limits its feasibility for routine assessment of disease severity during asthma exacerbations in clinical practice [24, 25]. However, the 3D-CT analysis platform SYNAPSE VINCENT (Fujifilm, Tokyo, Japan)—which is widely implemented in routine clinical settings and used in the present study—enables automated and quantitative evaluation of bronchial endpoint counts and bronchial volumes, thereby minimizing operator-dependent variability [11, 26]. Consequently, when chest CT equipment and this software are both available, objective and reproducible assessments can be conducted, even by non-specialists. Previous investigations have revealed that reductions in bronchial tree representations identified using 3D-CT analysis correlate with asthma severity [27]. The key question of this study was whether quantitative airway parameters derived from semi-automated three-dimensional CT analysis can identify structural airway abnormalities—particularly mucus plugging and distal airway obstruction—that underlie physiological impairment during asthma exacerbations. We aimed to evaluate their potential clinical utility as objective structural markers to complement physiological assessment, especially in patients with severe exacerbations requiring hospitalization, when CT is clinically indicated.

## Methods

### Study population

This exploratory, single-center, retrospective observational study was conducted among 23 patients with asthma aged 29–87 years. Adult patients with physician-diagnosed bronchial asthma who underwent chest CT during the study period were systematically screened using hospital medical records and the database. The observation period spanned from January 2010 to September 2024. The median interval between stable and exacerbation CT scans was 436 days (IQR, 305–1116 days). The inclusion criteria were as follows: (1) aged ≥ 18 years and diagnosed with bronchial asthma, (2) participation based on the patient’s free will with adequate understanding, (3) chest CT performed during both the stable and exacerbation phases of asthma, with 3D-CT analysis conducted using SYNAPSE VINCENT, and (4) CT scans acquired using comparable imaging conditions between the stable and exacerbation phases, including inspiration breath-hold, supine position, and a slice thickness of 1 mm. We excluded patients with interstitial pneumonia, lung cancer, pulmonary sequestration, situs inversus, bronchiectasis, pneumonia, or heart failure. After applying these criteria, 23 patients were included in the final analysis. The patient selection process is summarized in Supplementary Fig. 1. In our institution, chest CT during exacerbations was performed based on clinical indications at the discretion of the attending physician, rather than for research purposes. This retrospective study was approved by the institutional ethics committee (approval number: RK-241112-8), and the requirement for written informed consent was waived and an opt-out approach was implemented.

### Definitions of asthma exacerbation and stable phase

Asthma exacerbation was defined as any of the following: the need for systemic or oral corticosteroids due to an asthma attack, a visit to the emergency department, or hospitalization. The stable phase was defined as the absence of any visits to the clinic in the 2 weeks before or after an asthma exacerbation, as determined by the attending physician, and no need for changes in treating bronchial asthma.

### Arterial blood gas analysis and oxygenation assessment

Arterial blood gas (ABG) analysis was performed in hospitalized patients during asthma exacerbation as part of routine clinical evaluation when assessment of oxygenation status was clinically indicated. Arterial blood samples were obtained from the radial or femoral artery using standard clinical techniques. No procedure-related adverse events were observed. The PaO₂/FiO₂ (P/F) ratio was calculated using measured arterial partial pressure of oxygen (PaO₂) and the corresponding fraction of inspired oxygen (FiO₂) at the time of sampling. Moreover, FiO₂ was determined based on room air conditions (FiO₂ = 0.21) or documented supplemental oxygen administration. Peripheral oxygen saturation (SpO₂) was measured using pulse oximetry at the time of ABG sampling as part of routine clinical assessment.

### 3D-CT analysis of bronchial endpoint count and bronchial volume

Chest CT was performed at full inspiration, from the apex to the base of the lungs, with a slice thickness of 1 mm. All scans were acquired with patients in the supine position using a single breath-hold at full inspiration. Patients were instructed by trained technologists to inhale maximally and hold their breath prior to image acquisition, and adequate end-inspiratory breath-holding was visually confirmed on the CT console monitor. Theseacquisition procedures were applied consistently in both the stable and exacerbation phases. The use of standardized full-inspiration volumetric CT with 1-mm slice thickness ensured sufficient image quality and comparability for quantitative airway analysis. The obtained images were analyzed using the 3D-CT analysis software SYNAPSE VINCENT, and bronchial endpoint count and bronchial volume were measured using both automatic and manual segmentation techniques. Bronchial volume was measured using manual segmentation, starting from the second-order branches of each pulmonary lobe, in accordance with a previous study [15]. “SYNAPSE VINCENT” refers to a dedicated 3D image analysis software and should be considered a single integrated platform name, distinct from other SYNAPSE products (such as PACS systems) and not a separate “VINCENT” platform alone.

Bronchial volume measurement was performed using a standardized semi-automated segmentation protocol. As shown in Supplementary Fig. 2, predefined anatomical landmarks at the entrance of each lobar bronchus were used as reference points. The airway lumen distal to the entrance of each lobar bronchus was included in the segmentation. Based on the extracted airway tree, the software automatically calculated the bronchial lumen volume. To minimize measurement variability, identical software settings and standardized segmentation rules were applied consistently across all cases. Interobserver reproducibility was assessed by independent measurements performed by two observers using the same protocol. Interobserver agreement was excellent, with an intraclass correlation coefficient (ICC) of 0.9997 using a two-way mixed-effects model with absolute agreement. Bland–Altman analysis demonstrated a mean difference of 0.499, with limits of agreement ranging from − 1.643 to 2.642, indicating minimal measurement variability.

The percentage change in the bronchial parameters between the stable and exacerbation phases was calculated using the formula: (stable − exacerbation) / stable × 100%. This value represents the relative percentage change normalized to the stable phase.

### Mucus plug scoring

Mucus plug scoring on chest CT was performed using a system based on bronchopulmonary segmental anatomy and interpreted by at least two pulmonologists trained in chest radiology. Each bronchopulmonary segment was assigned a mucus plug score of 0 (absent) or 1 (present), and the total score was calculated as the sum of the segments with mucus plugs, ranging from 0 to 18 [25]. Lung areas within 2 cm of the pleura or diaphragm, extending toward the central bronchi, were excluded from the evaluation. Because the assessment was based solely on CT imaging, alternative causes of airway narrowing, such as focal bronchoconstriction or marked inflammatory airway wall edema, cannot be completely excluded. Therefore, to minimize potential misclassification, only lesions showing typical intraluminal soft-tissue attenuation completely occluding a segmental or subsegmental bronchus—consistent with the representative examples illustrated in the figures—were classified as mucus plugs.

Mucus plugs were defined according to the predefined criteria based on representative CT reference images (Supplementary Fig. 3A, B). Scoring was performed independently by two pulmonologists trained in chest radiology using a standardized scoring protocol. If any discrepancy was identified, even by a single point, the images were re-evaluated and a consensus score was determined through joint review. To assess interobserver reliability, intraclass correlation coefficient (ICC) and Bland–Altman analysis were performed based on the independently obtained measurements prior to consensus. Interobserver reproducibility of the mucus plug score was good. The intraclass correlation coefficient was 0.764 during the stable phase and 0.853 during exacerbations. Bland–Altman analysis demonstrated minimal bias with acceptable limits of agreement, indicating good interobserver agreement and minimal measurement variability.

### Statistical analyses

The data are summarized as median [interquartile range], and normality was assessed using the Shapiro–Wilk test. For non-normally distributed data, non-parametric methods were used for statistical analysis. To evaluate the relationship between variables, the Pearson correlation coefficient was applied if normality was confirmed, and the Spearman rank correlation coefficient was used if normality was not confirmed. All statistical analyses were performed using IBM SPSS Statistics for Windows, version 29 (IBM Corp., Armonk, NY, USA). Statistical significance was set at α = 0.05 (two-tailed), with *P* < 0.05 considered statistically significant.

## Results

### Patient characteristics

The baseline characteristics of the study population are presented in Table 1; among the 23 patients, eight (34.8%) were men. The biomarkers of type 2 inflammation were elevated, with a mean peripheral blood eosinophil count of 181.3/µL, fractional exhaled nitric oxide of 39 ppb, and total serum immunoglobulin E level of 294 IU/mL. During the stable phase, the mean daily dose of inhaled corticosteroids, expressed as fluticasone propionate equivalents, was 640 µg. Oral corticosteroids were used by 34.8% (*n* = 8) of patients, and 21.7% (*n* = 5) were receiving biologic therapy, which indicates a relatively high treatment intensity. Despite this, spirometric data revealed a mean FEV1 of 1.5 L, an FEV1/forced vital capacity ratio of 66.4%, and a V50/V25 ratio of 3.7, which is indicative of persistent airway obstruction.

**Table 1:** Baseline characteristics (*n* = 23)

Demographics	
Age (years)	69 [29, 87]
Male sex	8 (34.8)
BMI (kg/m^2^)	22.2 [18.0, 52.7]
Smoking status	
Ever smoker	6 (26.1)
Current smoker	4 (17.4)
Complications	
Atopic dermatitis	3 (13.0)
Allergic rhinitis	8 (34.8)
Medication	
Inhaler	19 (82.6)
ICS/LABA/LAMA	3 (13.0)
ICS/LABA	16 (69.6)
ICS dose (FP equivalent dose, µg/day)	640 [0, 960]
LAMA	6 (26.1)
LTRA	12 (52.2)
Theophylline	8 (34.8)
OCS	8 (34.8)
PSL dose	0 [0, 10]
History of biologics use	5 (21.7)
Biomarkers of type 2 inflammation	
Blood eosinophil count (cells/µL)	181.3 [0, 2340]
FeNO (ppb)	39.0 [5, 167]
IgE (IU/mL)	294.0 [0, 8922]
Functional outcomes	
FEV1 (L)	1.5 [0.8, 3.1]
FEV1 (% pred)	94.1 [35.4, 169.9]
FEV1/FVC (%)	66.4 [30.3, 87.6]
VC (L)	3.0 [1.3, 4.2]
VC (% pred)	117.5 [68.8, 142.3]
FVC (L)	2.8 [1.3, 4.0]
V50/V25	3.7 [2.1, 7.3]
Mucus plug score	0 [0, 10]

Values are presented as median (range) or number (percentage), as appropriate

*Abbreviations*: *BMI *body mass index,  *FeNO* fractional exhaled nitric oxide, *FEV1 *forced expiratory volume in 1 s, *FVC* forced vital capacity,  *FP *fluticasone propionate, *ICS *inhaled corticosteroid, *IgE *immunoglobulin,  *E LABA* long-acting beta2-agonist, *LAMA *long-acting muscarinic antagonist, *LTRA *leukotriene receptor antagonist, *OCS *oral corticosteroid, *PSL *prednisolone, *VC *vital capacity, V50/V25 maximum expiratory flow rate at 50% of vital capacity/maximum expiratory flow rate at 25% of vital capacity

### Changes in bronchial endpoint count and bronchial volume during asthma exacerbations

**Fig. 1:**
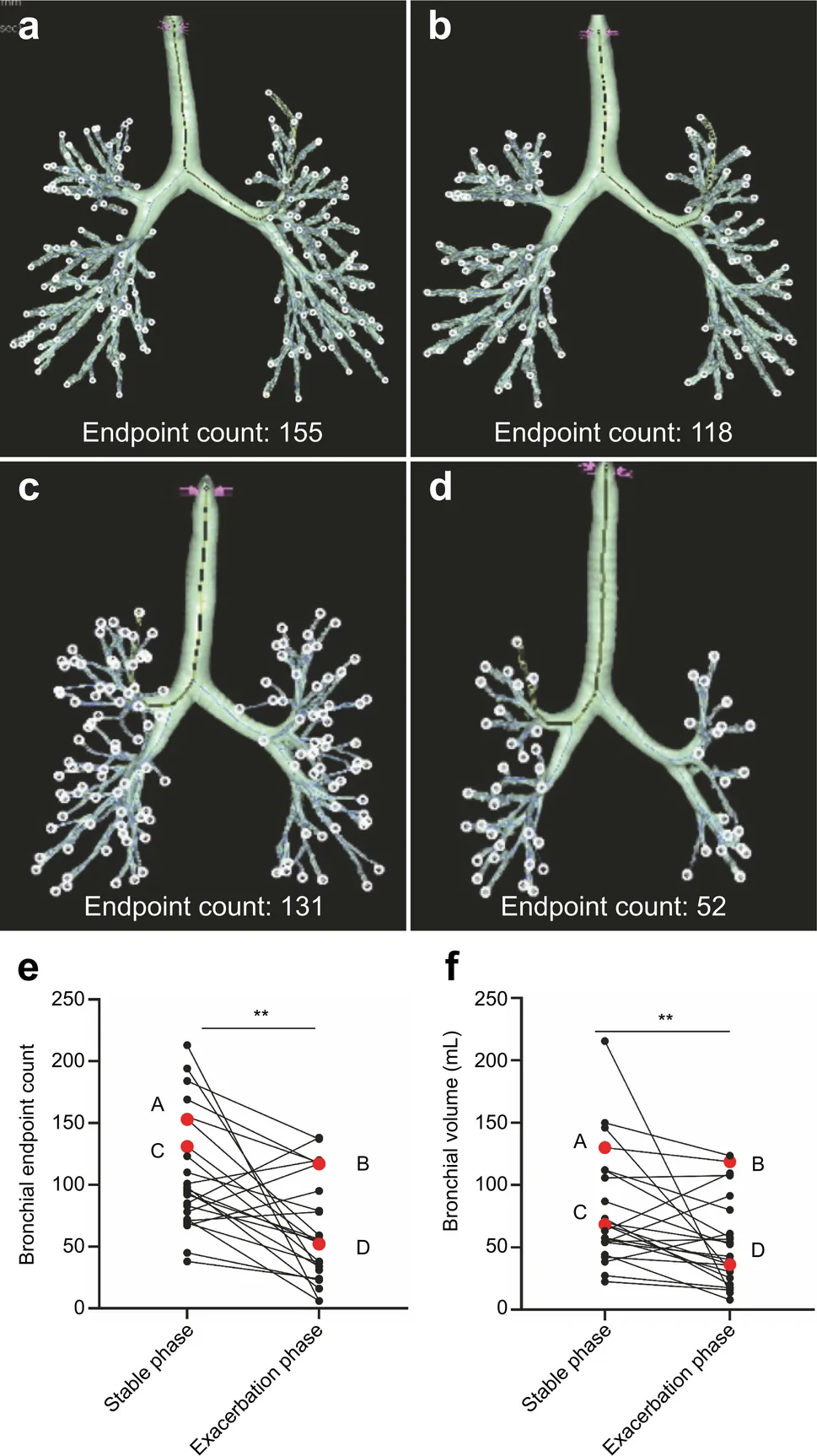
3D-CT visualization of airway changes in stable and exacerbation phases of asthma. (**a**-**d**) Representative 3D-CT images of the bronchial tree in two patients with asthma: patient 1 in the stable (**a**) and exacerbation (**b**) phases; patient 2 in the stable (**c**) and exacerbation (**d**) phases. The bronchial endpoint count was lower in the exacerbation phase in both patients. The endpoint counts were as follows: patient 1: (**a**) 155 and (**b**) 118; patient 2: (**c**) 131 and (d) 52. (**e**) Comparison of the bronchial endpoint counts between the stable and exacerbation phases (paired t-test: P < 0.05). Red points labeled A–D correspond to the representative cases shown in panels (**a**–**d**), respectively. (**f**) Comparison of the bronchial volume between the two phases (Wilcoxon signed-rank test: P < 0.01). Red points labeled A–D correspond to the representative cases shown in panels (**a**–**d**), respectively. 3D-CT, three-dimensional computed tomography.

3D-CT analysis was performed to compare the bronchial morphology between the stable and exacerbation phases of asthma. As shown in Fig. 1, the number of bronchial branches visualized on 3D reconstructions was markedly lower during exacerbations (Fig. 1b, d) than during the stable phase in both the represented individuals (Fig. 1a, c).

Quantitative analysis revealed a significantly lower bronchial endpoint count (Fig. 1e) and bronchial volume (Fig. 1f) during exacerbations than during the stable phase.

Clinical characteristics during exacerbation are summarized in Supplementary Table 1. All patients presented with worsening respiratory symptoms and wheeze on chest auscultation and were diagnosed with asthma exacerbation. Nearly all patients received systemic corticosteroids administered intravenously, and all patients required treatment escalation. Overall, 65% of patients required hospitalization. Blood eosinophil counts during exacerbation were relatively low, which may reflect the effects of biologic therapy or corticosteroid treatment. FeNO measurements were not performed during exacerbation due to respiratory distress.

In addition, we conducted a subgroup analysis comparing patients with and without a smoking history and found no significant differences in the bronchial endpoint count or bronchial volume between these groups(Supplementary Fig. 4).

### Correlation of mucus plug score with bronchial endpoint count and bronchial volume

**Fig. 2:**
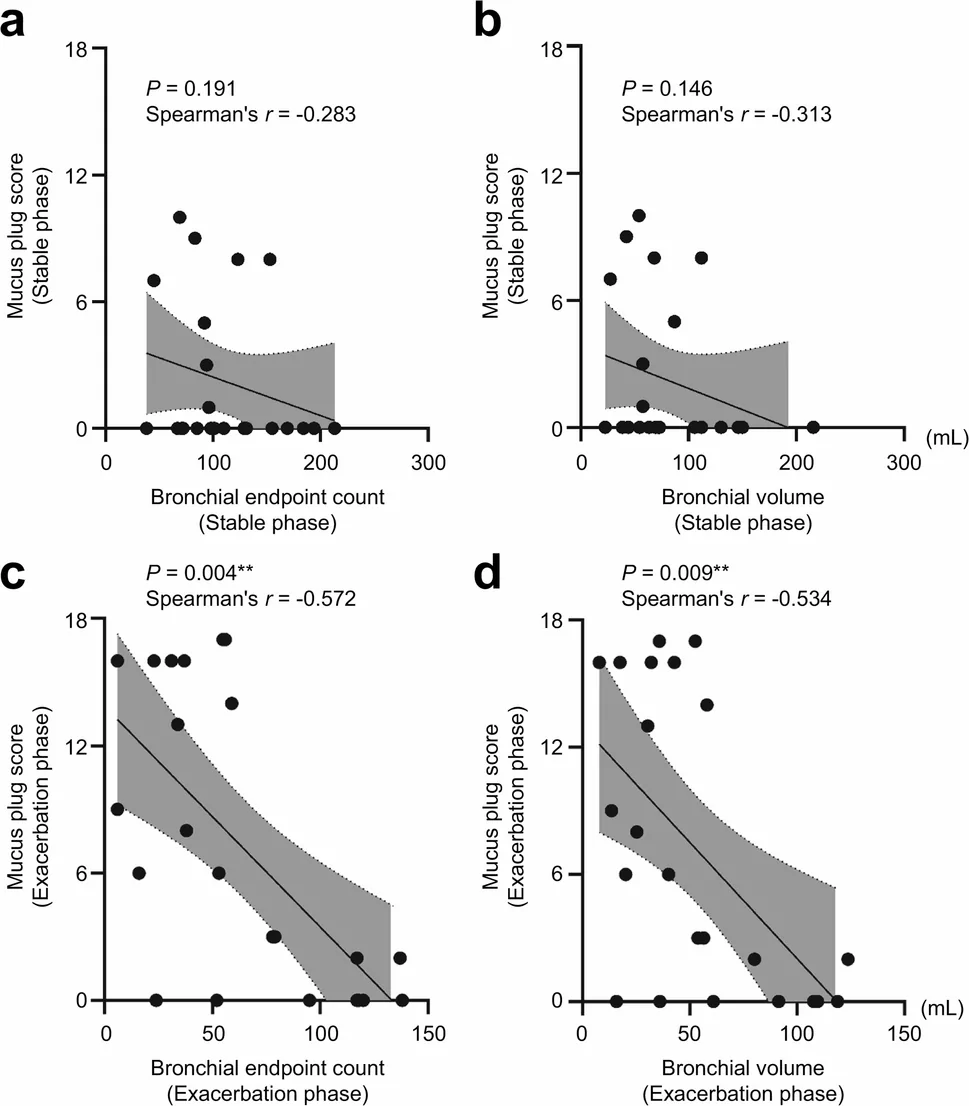
Correlation between the mucus plug score and airway parameters in the stable and exacerbation phases. Correlation of the mucus plug score with bronchial endpoint count and volume during the stable (**a**, **b**) and exacerbation (**c**, **d**) phases. No significant correlations were found during the stable phase (**a**, **b**). A significant negative correlation was observed during exacerbation between the mucus plug score and both the bronchial endpoint count (Spearman’s r = −0.572, P = 0.004) (**c**) and bronchial volume (Spearman’s r = −0.534, P = 0.009) (**d**). Shaded gray areas represent 95% confidence intervals

Given the recognized role of mucus plugs in contributing to airway narrowing during asthma exacerbations, we examined the association between mucus plug burden and quantitative airway metrics derived from 3D-CT. Mucus plug score was not significantly correlated with bronchial endpoint count or bronchial volume during the stable phase; however, during exacerbations, a significant inverse correlation was found with both bronchial endpoint count and bronchial volume (Fig. 2a, c for endpoint count; Fig. 2b, d for bronchial volume).

### Correlation between mucus plug score and percentage change of bronchial endpoint count and bronchial volume

**Fig. 3:**
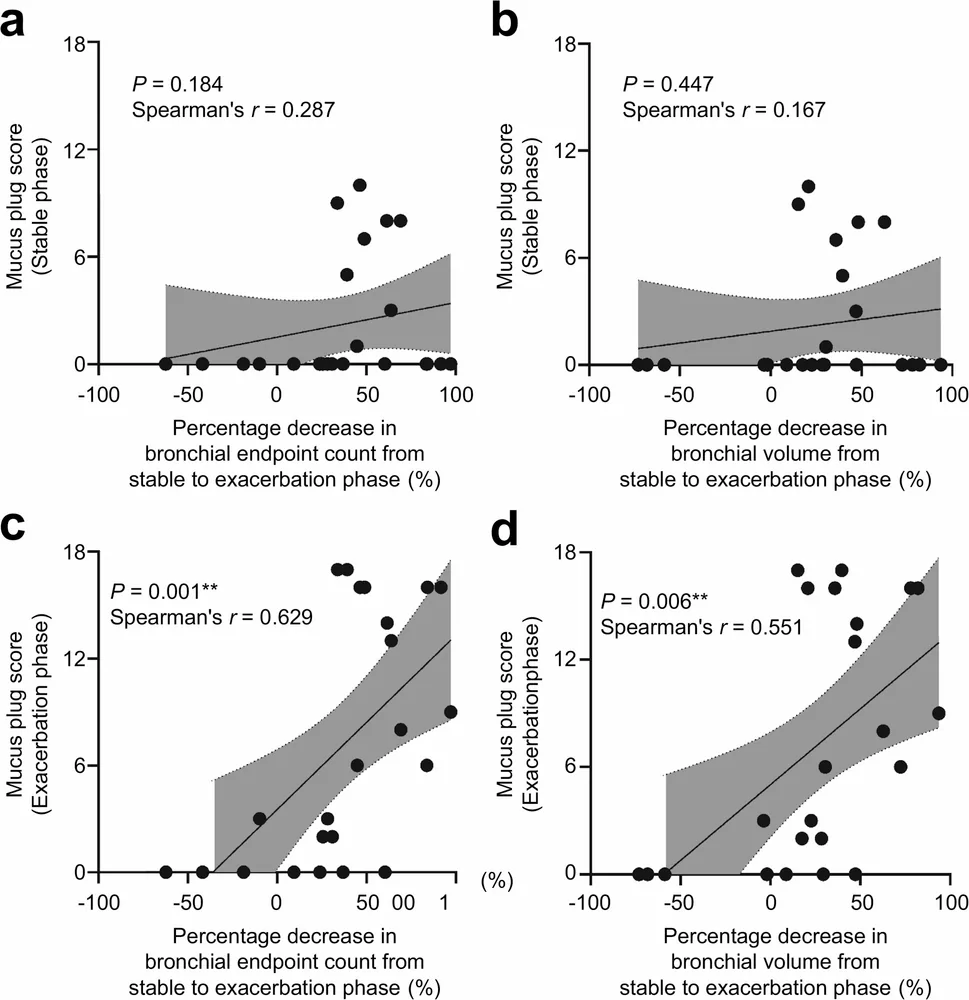
Correlation between mucus plug score and percentage change in airway parameters. Correlation between the mucus plug score and the percentage change in bronchial endpoint count (**a**, **c**) and volume (**b**, **d**) from the stable to exacerbation phase. No significant correlations were found during the stable phase (**a**, **b**). Significant positive correlations were observed during the exacerbation phase between the mucus plug score and both the percentage change of the bronchial endpoint count (Spearman’s r = 0.629, P = 0.001) (**c**) and the percentage change of bronchial volume (Spearman’s r = 0.551, P = 0.006) (**d**). Shaded gray areas represent 95% confidence intervals

We further analyzed whether the mucus plug score was associated with the rates of reduction in bronchial endpoint count and bronchial volume from the stable to exacerbation phase. During the stable phase, no significant correlation was observed between mucus plug score and the percentage change of either bronchial endpoint count or bronchial volume. Conversely, during exacerbations, a higher mucus plug score was significantly correlated with the percentage change of bronchial endpoint count (Fig. 3a, c) and bronchial volume (Fig. 3b, d).

### Correlations of oxygenation indices with bronchial endpoint count and bronchial volume during hospitalization for exacerbation

ABG parameters and SpO₂ values during exacerbation are summarized in Supplementary Table 2.

**Fig. 4:**
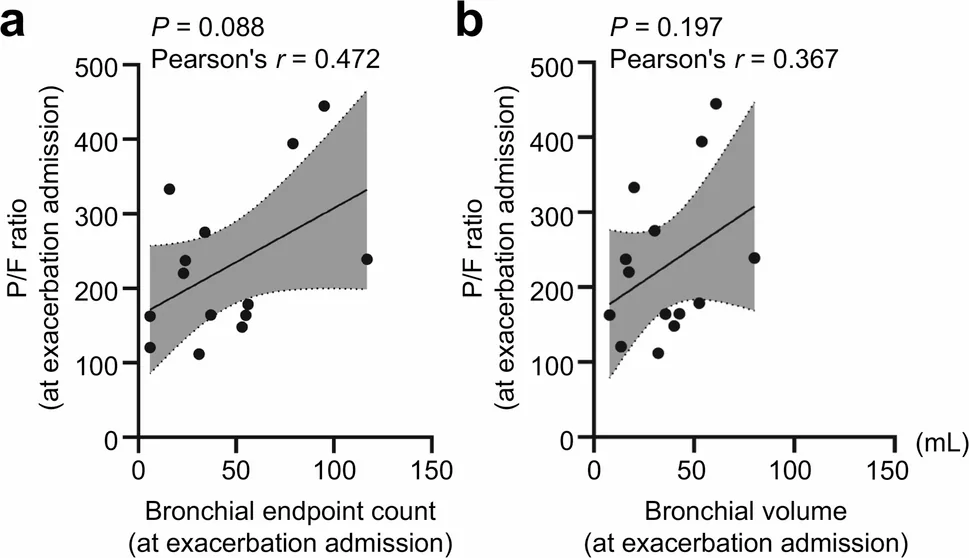
Correlation between oxygenation and absolute airway parameters during exacerbation. Correlation of PaO2/FiO2 ratio at the time of exacerbation-related hospitalization with bronchial endpoint count (**a**) and volume (**b**). No significant correlations were observed: (**a**) Pearson’s r = 0.472, P = 0.088; (**b**) Pearson’s r = 0.367, P = 0.197. Shaded gray areas represent 95% confidence intervals

To explore the relationship between airway structural changes and oxygenation status during asthma exacerbations, we analyzed the correlation between the PaO_2_/FiO_2_ (P/F) ratio and both bronchial endpoint count and bronchial volume measured at the time of hospitalization. Neither bronchial endpoint count nor bronchial volume during the exacerbation phase was significantly correlated with the P/F ratio (Fig. 4a, b).

### Correlations of oxygenation indices with percentage change of bronchial endpoint count and bronchial volume during hospitalization for exacerbation

**Fig. 5:**
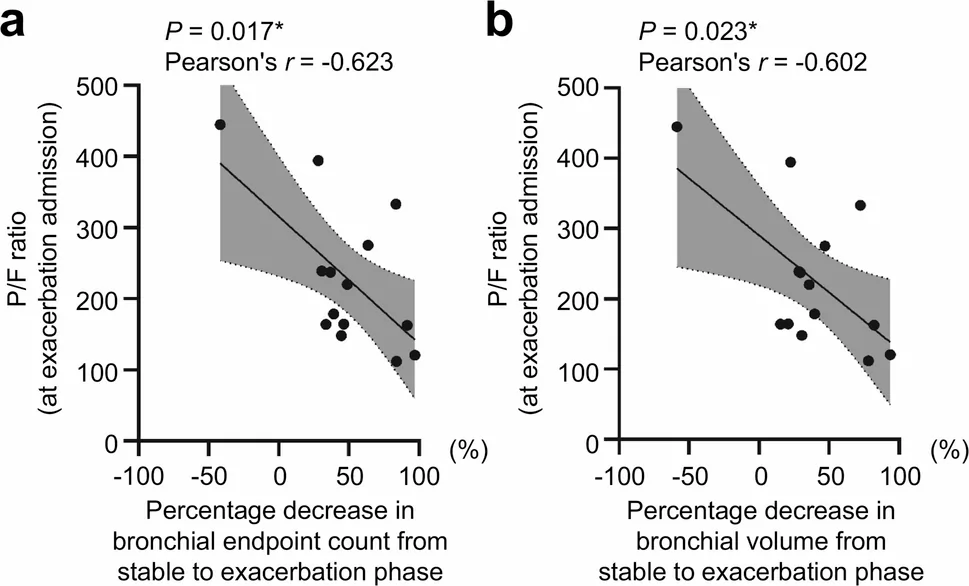
Correlation between oxygenation and percentage change in airway parameters. Correlation of PaO2/FiO2 ratio at the time of exacerbation-related hospitalization and percentage change (from the stable phase) in bronchial endpoint count (**a**) and bronchial volume (**b**). Significant correlations were found: (**a**) Pearson’s r = −0.623, P = 0.017; (b) Pearson’s r = −0.602, P = 0.023. Shaded gray areas represent 95% confidence intervals

To investigate the relationship between dynamic airway structural changes and oxygenation status during asthma exacerbations, we assessed the correlation between the P/F ratio during hospitalization and the percentage change of bronchial endpoint count and bronchial volume from the stable to exacerbation phase. Significant negative correlations were observed between the P/F ratio and the percentage change of bronchial endpoint count (Fig. 5a) and bronchial volume (Fig. 5b) during hospitalization for exacerbation. These findings suggest that it is not the static airway structure at a single time point but rather the magnitude of structural deterioration from the stable phase—as reflected by the percentage change in bronchial parameters—that most closely reflects impairment in oxygenation. Therefore, quantitative 3D-CT analysis of airway changes over time may provide clinically meaningful insights into the physiological consequences of acute asthma exacerbations.

## Discussion

Although 3D-CT offers detailed visualization of organ morphology, its clinical application in managing asthma remains limited, and it is still undergoing early-stage integration into routine practice. Herein, we quantitatively assessed airway structural changes during asthma exacerbations using bronchial endpoint count and bronchial volume measurements derived from 3D-CT analysis.

Bronchial endpoint count and bronchial volume were significantly lower during exacerbations than during the stable phase. These findings suggest that asthma exacerbations are accompanied by measurable structural airway changes that can be quantitatively detected using 3D-CT analysis. Furthermore, both the absolute values and percentage changes in these airway parameters significantly correlated with the mucus plug scores during exacerbations. These associations indicate that reductions in the bronchial endpoint count and bronchial volume may reflect airway narrowing associated with mucus obstruction. Although the bronchial endpoint count and bronchial volume measured at a single time point during exacerbations were not significantly associated with the P/F ratio, their percentage change from the stable phase were significantly correlated with the oxygenation indices. This finding suggests that dynamic structural changes, rather than static measurements alone, may be more closely associated with physiological impairment during exacerbations.

To the best of our knowledge, this is the first study revealing that these quantitative parameters are significantly associated with both mucus plug burden and oxygenation impairment based on ABG analysis.

Importantly, the 3D-CT analysis platform used in this study is currently available in routine clinical settings. Moreover, its ability to provide rapid, quantitative, and examiner-independent assessments supports its potential utility as an objective tool for evaluating the severity of asthma exacerbations.

In patients with asthma, reductions in the number of visible bronchi are believed to reflect both chronic inflammation–induced airway remodeling and mechanical obstruction of the peripheral airways by mucus plugs [27, 28]. Notably, intraluminal mucus accumulation has been implicated in the exacerbation of airflow limitation, and contributes to ventilation heterogeneity and impaired gas exchange. The association between mucus plugs and regional ventilation abnormalities has garnered increasing attention [29].

However, assessing mucus plugs remains challenging and represents a key barrier to routine clinical application [14, 30, 31]. Conventional mucus plug scoring requires specialized expertise for accurate interpretation of chest CT images and involves a labor-intensive, time-consuming evaluation process [14, 32]. The 3D-CT analysis system employed in this study, SYNAPSE VINCENT, enables near-automated quantification of bronchial endpoint counts and bronchial volume, thereby minimizing operator dependency and enhancing reproducibility [11, 26]. Accordingly, when chest CT and this system are both available, objective evaluations can be conducted, even by non-specialist clinicians. While previous studies in chronic obstructive pulmonary disease have revealed associations between bronchial count, airway wall area, and mucus plug burden as assessed using 3D-CT, to the best of our knowledge, the relationship between bronchial endpoint count, bronchial volume, and mucus plugs specifically during asthma exacerbations has not been investigated [33]. This study is the first to demonstrate that both the bronchial endpoint count during exacerbation and its percentage change from the stable phase are significantly correlated with mucus plug scores, based on automated quantification using a 3D-CT analysis platform. Furthermore, not only reductions in bronchial endpoint count but also decreases in bronchial volume were significantly associated with mucus plug burden during exacerbations.

A key finding from this study is that the percentage change of bronchial endpoint count and intraluminal bronchial volume were significantly correlated not only with mucus plug burden but also with oxygenation status during asthma exacerbations. The clinical severity of asthma exacerbations is typically evaluated based on subjective symptoms, respiratory rate, physical examination findings (e.g., auscultatory abnormalities), and the degree of respiratory failure assessed using arterial blood gas analysis [8, 9]. However, reliable imaging-based objective markers for exacerbation severity have yet to be established. Furthermore, conventional assessment approaches are inherently subjective and prone to inter-observer variability, which is frequently influenced by differences in clinical expertise and judgement [34, 35]. ABG analysis remains a standard clinical tool for assessing oxygenation and provides a functional indicator of gas exchange impairment at a given time point. However, ABG analysis does not identify the structural causes underlying oxygenation impairment. In contrast, quantitative 3D-CT airway analysis enables objective assessment of structural airway abnormalities that may contribute to impaired ventilation and gas exchange, including airway narrowing, distal airway obstruction, reduced visibility of peripheral airways, and mucus impaction. When oxygenation is impaired during asthma exacerbations, clinical management requires not only recognition of physiological severity but also identification of the underlying cause to guide appropriate intervention. In this context, quantitative 3D-CT airway analysis is clinically relevant because it can objectively detect and quantify structural airway abnormalities that may contribute to respiratory deterioration, particularly mucus plugging and distal airway obstruction.

In this study, quantitative airway parameters derived from semi-automated 3D-CT analysis, including bronchial endpoint count and bronchial volume, were significantly associated with oxygenation indices. These findings support the concept that structural airway abnormalities quantified by 3D-CT contribute to oxygenation impairment during exacerbations and indicate the potential clinical utility of 3D-CT airway analysis for evaluating the structural basis of respiratory deterioration. We do not propose CT as a routine tool for stable asthma or for all exacerbations. Rather, in select patients with severe exacerbations requiring hospitalization and impaired oxygenation, when CT is clinically indicated, quantitative 3D-CT airway analysis may provide clinically actionable information by identifying treatable airway obstruction, such as mucus plugging and distal airway abnormalities, and thereby support treatment-oriented clinical evaluation. In addition, quantitative 3D-CT analysis provides an objective research tool for characterizing airway structural changes during exacerbations. Given the exploratory nature of this study and its relatively small sample size, these findings should be interpreted cautiously and require further validation in prospective studies.

Despite these promising clinical implications, radiation exposure remains an important consideration when applying CT-based airway analysis. In the present study, CT examinations were performed only when clinically indicated, such as during severe exacerbations requiring hospitalization or when alternative diagnoses needed to be excluded. The median interval between stable-phase and exacerbation-phase CT scans was 436 days (IQR, 305–1116 days), indicating that CT imaging was performed infrequently. Therefore, the cumulative radiation exposure associated with this approach is expected to be limited. Importantly, CT-based airway analysis does not require additional imaging, as it can be performed using clinically indicated CT scans. Thus, this approach may provide valuable structural information in selected clinical scenarios while minimizing additional radiation burden. This study had some limitations. First, it was conducted in a single center and had a relatively small sample size, which may limit the generalizability of the findings. In addition, the small sample size may have limited the robustness of the regression analyses. Although statistically significant associations were observed, the coefficients of determination (R²) were relatively small, indicating limited explanatory power of the models. Furthermore, the possibility that individual data points may have disproportionately influenced the regression results cannot be excluded. Therefore, these findings should be interpreted with caution.

Larger, multicenter studies are warranted to validate these results in broader clinical populations. Secondly, we did not include a comparison group of patients with milder asthma who had not experienced exacerbations. Such a group would have allowed a clearer distinction between structural changes specifically associated with exacerbations and those reflecting baseline disease severity. Future prospective studies including patients with milder asthma without exacerbations are warranted to better define the specificity of 3D-CT–derived airway changes observed during exacerbations.

Third, although we retrospectively investigated the association between 3D-CT–derived airway parameters and hospitalization during asthma exacerbations, the extent to which 3D-CT–based assessments influence clinical decision-making in real time remains unclear. Prospective, multicenter investigations are needed to determine whether incorporating 3D-CT airway analysis into clinical workflows improves patient management and outcomes. Furthermore, because this study had a retrospective design, objective measures of disease stability, such as peak expiratory flow monitoring, symptom scores, or asthma control questionnaires, were not systematically available. Therefore, some patients classified as stable may have had subclinical disease activity. This limitation should be considered when interpreting the observed differences between stable and exacerbation phases.

In addition, chest CT during exacerbations was performed based on clinical indications rather than routinely in all patients. Therefore, the study population may represent a selected subset of patients, which may limit the generalizability of the findings.

Fourth, differences in the inspiratory effort between stable and exacerbation phases may influence airway visibility and quantitative airway measurements. Although CT scans were acquired with standardized breath-hold instructions and no significant difference in the total lung volume was observed between the phases in our analysis, subtle differences in the inspiratory level cannot be completely excluded in this retrospective study　(Supplementary Fig. 5). Reduced inspiratory effort during exacerbation could theoretically decrease peripheral airway visibility and segmentation, leading to underestimation of bronchial endpoint count and bronchial volume independent of true structural changes. Therefore, any residual variability in the inspiratory level would most likely bias the results toward underestimation of airway dimensions during exacerbation. Furthermore, because this study was conducted over an extended period in a real-world clinical setting, standardization of CT scanner models was not feasible owing to clinical and logistical constraints. However, all CT scans included in the analysis met the predefined acquisition criteria for quantitative airway assessment, including helical acquisition with a slice thickness of 1 mm during a single breath-hold at full inspiration, with adequate inspiratory effort visually confirmed on the CT console monitor. These standardized imaging conditions likely ensured sufficient spatial resolution and comparability required for accurate airway segmentation and quantitative analysis. Nevertheless, as this was a retrospective study, the potential influence of scanner-related variability cannot be completely excluded and should be considered when interpreting the results.

Fifth, although the causal relationship between mucus plugs and physiological impairment has not been fully established, our additional analysis demonstrated that the number of mucus plugs per bronchus was limited. However, even a single mucus plug completely occluding a segmental bronchus may render the distal lung unit non-ventilated and contribute to gas exchange impairment through regional ventilation–perfusion mismatch, as supported by prior studies [24, 36]. Therefore, while this study does not directly establish causality, mucus plugs represent clinically meaningful structural abnormalities associated with physiological impairment.

Sixth, although the P/F ratio is a well-established physiological indicator of oxygenation impairment, it may be influenced by multiple factors beyond airway structure alone. Importantly, ABG analysis provides the primary physiological assessment of respiratory impairment, whereas CT airway analysis serves to evaluate the structural contributors underlying the observed physiological abnormalities. Therefore, the association between CT-derived airway parameters and oxygenation indices in this study should be interpreted as reflecting structural correlates of physiological impairment rather than direct causality. Future prospective studies incorporating standardized physiological and imaging assessments are warranted to further establish the clinical utility of CT-derived airway parameters.

## Conclusions

In this study, we provide novel evidence that clinically available semi-automated quantitative 3D-CT analysis of airway structures can objectively capture pathophysiological changes during asthma exacerbations. While neither bronchial endpoint count nor intraluminal bronchial volume alone was associated with oxygenation status, their respective percentage change from the stable phase were significantly correlated with the P/F ratio, which indicates a dynamic structural basis for impairments in gas exchange. In contrast, both absolute values and percentage change were significantly correlated with mucus plug burden during the exacerbation phase. These findings highlight the utility of 3D-CT–derived reduction metrics as surrogate markers that reflect not only airway obstruction by mucus plugs but also functional consequences, including impaired oxygenation. Given that 3D-CT is a non-invasive, rapid, and examiner-independent method, it holds promise as an objective imaging-based tool for evaluating the severity of asthma exacerbations and guiding clinical decision-making. Prospective multicenter studies are warranted to validate these findings and determine their impact on patient outcomes in routine practice.

## Supplementary Information

Supplementary Material 1.

Supplementary Material 2.

## Data Availability

The data that support the findings of this study are available from the corresponding author (KM) upon reasonable request. The individual participant data collected for this study will not be shared, as the data contain potentially identifiable information, and no consent for data sharing was provided.

## Abbreviations

CT: Computed tomography
FEV1: Forced expiratory volume in 1 s
P/F ratio: PaO_2_/FiO_2_ ratio
3D-CT: Three-dimensional computed tomography
