# Supplementary material for: 3D CT–Based quantification of airway structural changes during asthma exacerbations: a retrospective observational study
Source: BMC Pulm Med. 2026 Jun 30;26:436. doi: 10.1186/s12890-026-04464-z (PMC13621706; doi:10.1186/s12890-026-04464-z)
Supplement: Supplementary file 1 [file 12890_2026_4464_MOESM1_ESM.docx]

**Supplementary Figures**

**Supplementary Figure 1. Flow diagram of patient selection.**

Flow diagram illustrating the patient selection process for this retrospective study. Adult patients with physician-diagnosed asthma who underwent chest computed tomography (CT) during the study period were screened. Patients meeting predefined exclusion criteria were excluded, including those with structural lung abnormalities, alternative causes of respiratory deterioration, CT examinations performed only during either the stable or exacerbation phase, and differences in the CT acquisition parameters. Patients who had undergone chest CT during both the clinically stable phase and an acute exacerbation were included in the final analysis (n = 23).

**Supplementary Figure 2. Semi-automated bronchial segmentation workflow.**

(A) Representative three-dimensional reconstruction of the bronchial tree from chest computed tomography (CT). The region used for bronchial volume measurement is indicated by a white dashed outline, corresponding to the airway lumen distal to the lobar bronchial entrances.

(B) Bronchial tree after semi-automated segmentation with manual refinement. Segmentation was performed based on predefined anatomical landmarks, and consistent software settings were applied across all cases.

**Supplementary Figure 3. Representative chest computed tomography (CT) images of mucus plugs in patients with asthma.**

(a, b) Representative chest CT images showing mucus plugs in segmental or subsegmental bronchi. Panel (a) shows a horizontally oriented mucus plug, while panel (b) shows a longitudinally oriented mucus plug along the bronchial axis. Mucus plugs were defined as intraluminal soft-tissue attenuation completely occluding the bronchial lumen. Arrows indicate representative mucus plugs.

**Supplementary Figure 4. Comparison of the airway measurements according to smoking history.**

The graphs compare the bronchial endpoint count (a) and bronchial volume (b) in patients with and without a smoking history. Vertical error bars represent 95% confidence intervals. Statistical comparisons were performed using an unpaired t-test for bronchial endpoint count and the Mann–Whitney U test for bronchial volume.

**Supplementary Figure 5. Comparison of computed tomography (CT)-derived total lung volume between stable and exacerbation phases.**

The graph compares the total lung volume measured from chest CT during the clinically stable and exacerbation phases. Total lung volume was calculated using CT-based volumetry with the SYNAPSE VINCENT software. Vertical error bars represent 95% confidence intervals. Statistical comparisons between the two phases were performed using a paired t-test.
