# Supplementary material for: 3D CT–Based quantification of airway structural changes during asthma exacerbations: a retrospective observational study
Source: BMC Pulm Med. 2026 Jun 30;26:436. doi: 10.1186/s12890-026-04464-z (PMC13621706; doi:10.1186/s12890-026-04464-z)
Supplement: Supplementary file 2 [file 12890_2026_4464_MOESM2_ESM.docx]

**Supplementary Tables**

**Supplementary Table 1.** Clinical characteristics of patients during asthma exacerbations.

| Patients with clinical symptoms | 23(100) |
| --- | --- |
| Patients with wheezing | 23(100) |
| Emergency department visit | 23(100) |
| Hospitalization | 15(65) |
| Treatment escalation | 23(100) |
| Systemic corticosteroid use | 22(95) |
| Blood eosinophil count (/μL) | 27.2[0,4930] |

Data are presented as number (%) or median [range], as appropriate. All patients presented with clinical symptoms and wheezing. Emergency department visits were required in all patients, and 65% required hospitalization. Treatment escalation was performed in all patients, and systemic corticosteroids were administered in 95% of cases. Blood eosinophil counts during exacerbation were relatively low, which may reflect the effects of biologic therapy or systemic corticosteroid treatment. Fractional exhaled nitric oxide (FeNO) measurements were not performed during exacerbation due to respiratory distress.

**Supplementary Table 2.** Arterial blood gas and oxygenation parameters during asthma exacerbations.

| Demographics |  |
| --- | --- |
| SpO_2_ (%) | 95.5 [87,100] |
| FiO_2_ | 0.31 [0.21, 1.00] |
| Receiving oxygen (%) | 85.7 |
| PH | 7.39 [7.24, 7.5] |
| PaCO_2_ (mmHg) | 41.8 [26.4, 70] |
| PaO_2_ (mmHg) | 74.95 [43.6, 112.0] |
| P/F | 199.3 [112.0, 444.8] |

This table summarizes peripheral oxygen saturation (SpO₂), fraction of inspired oxygen (FiO₂), arterial pH, partial pressure of arterial carbon dioxide (PaCO₂), partial pressure of arterial oxygen (PaO₂), and the PaO₂/FiO₂ (P/F) ratio measured during asthma exacerbations. Arterial blood gas analysis was performed as part of routine clinical assessment. Supplemental oxygen was administered in 85.7% of patients at the time of measurement, with a median FiO₂ of 0.31. Despite oxygen administration, the median P/F ratio was 199, indicating persistent impairment in gas exchange. The P/F ratio was included as a standardized physiological indicator of oxygenation severity, allowing evaluation of the clinical relevance of computed tomography-derived airway structural parameters in relation to physiological respiratory impairment.
